# Cardiovascular Toxicity of Novel HER2-Targeted Agents and Multikinase Inhibitors in Oncology: From Mechanisms to Real-World Clinical Evidence

**DOI:** 10.3390/ph19060833

**Published:** 2026-05-27

**Authors:** Amro Abu Suleiman, Vincenzo Quagliariello, Luigi Spadafora, Federico Russo, Nicola Maurea

**Affiliations:** 1 Department of Internal Medicine, York and Scarborough Teaching Hospitals NHS Trust, York YO31 8HE, UK; amro.abu-suleiman@nhs.net; 2 Division of Cardiology, Istituto Nazionale Tumori IRCCS Fondazione Giovanni Pascale, 80131 Naples, Italy; v.quagliariello@istitutotumori.na.it; 3 Department of Medical-Surgical Sciences and Biotechnologies, Sapienza University of Rome, 04100 Latina, Italy; fedrusso1@gmail.com; 4 Division of Cardiology, Sant’Andrea University Hospital, 00189 Rome, Italy

**Keywords:** cardio-oncology, cardiotoxicity, HER2, targeted therapy, heart failure, trastuzumab, tyrosine kinase inhibitors

## Abstract

The advent of novel Human Epidermal growth factor Receptor 2 (HER2)-targeted therapies and tyrosine kinase inhibitors (TKIs) has significantly improved outcomes in HER2-positive malignancies, particularly breast cancer. However, these agents carry a growing burden of cardiovascular adverse events, representing a critical concern in modern oncology. This narrative review explores the evolving landscape of cardiovascular toxicity associated with these therapeutic classes, integrating mechanistic insights with real-world clinical data. HER2-targeting monoclonal antibodies and antibody–drug conjugates exert off-target effects on cardiomyocytes via HER2 pathway inhibition, leading to reversible or irreversible myocardial dysfunction. In parallel, small-molecule TKIs, especially those targeting multiple kinases, have been associated with hypertension, arrhythmia, QT prolongation, and heart failure, through mechanisms such as mitochondrial dysfunction, endothelial damage, and disruption of cardioprotective signaling. We summarize clinical evidence elucidating the molecular basis of these toxicities and critically review clinical trials and post-marketing data highlighting their incidence and management. The review emphasizes the heterogeneity of cardiotoxicity profiles across different agents, underscoring the need for individualized cardiovascular risk stratification and monitoring. Finally, we address the emerging role of cardio-oncology in bridging oncologic efficacy with cardiac safety, advocating for multidisciplinary approaches, biomarker-guided surveillance, and standardized definitions of cardiotoxicity. As precision oncology advances, a parallel refinement in cardiotoxicity prediction and prevention is imperative to optimize patient outcomes.

## 1. Introduction

Human epidermal growth factor receptor 2 (HER2) overexpression is a well-established oncogenic driver implicated in malignancy, most notably breast cancer, as well as gastric, lung, and colorectal cancers [1]. The advent of HER2-targeted therapies, including monoclonal antibodies, antibody–drug conjugates (ADCs), and tyrosine kinase inhibitors (TKIs) has significantly transformed the therapeutic landscape leading to improved survival rates and prolonged disease control [2]. As these agents continue to be integrated into earlier lines of treatment and broader oncologic indications, understanding their safety profile has become increasingly critical. Among the emerging safety concerns, cardiovascular toxicity has gained particular attention as a clinically meaningful and potentially treatment-limiting adverse effect [3,4,5]. HER2 signaling is known to play a vital role in cardiomyocyte survival and stress adaptation [6,7,8,9]; thus, pharmacologic inhibition may result in on-target cardiac dysfunction.

Moreover, several TKIs—especially those targeting multiple kinases—exert off-target effects that contribute to a spectrum of cardiovascular toxicities, including hypertension, arrhythmias, left ventricular dysfunction, and ischemic events. These complications are further amplified in the real-world oncology setting, where patients often present with comorbidities and polypharmacy.

Given the increasing use of HER2-targeted therapies and TKIs across tumor types, a comprehensive understanding of their cardiovascular safety is urgently needed. This narrative review aims to: (i) elucidate the underlying pathophysiological mechanisms of cardiotoxicity associated with these agents; (ii) summarize the current clinical trial and real-world evidence regarding incidence, severity, and reversibility of cardiovascular events; and (iii) highlight the role of cardio-oncology in optimizing surveillance and multidisciplinary management strategies.

## 2. Methods

This study was conducted as a narrative review aimed at summarizing and critically discussing the current evidence on the cardiovascular toxicity associated with novel HER2-targeted agents and multikinase inhibitors used in oncology. The review focused on integrating mechanistic insights, clinical trial data, and real-world evidence in order to provide a clinically relevant overview of this emerging field.

A literature search was performed using PubMed/MEDLINE as the primary biomedical database. To broaden the scope of the review and reduce the risk of omitting relevant literature, supplementary searches were also performed using Scopus, Web of Science, and Google Scholar. Additional references were identified through manual screening of the bibliographies of selected articles, pivotal clinical trials, meta-analyses, international guidelines, and expert consensus statements. Given the narrative nature of this review, the search was not intended to be exhaustive in the manner of a systematic review, and no formal risk-of-bias assessment or quantitative synthesis was undertaken.

The search strategy included combinations of the following keywords and Medical Subject Headings (MeSH) terms: “HER2-targeted therapy”, “trastuzumab”, “pertuzumab”, “trastuzumab emtansine”, “trastuzumab deruxtecan”, “lapatinib”, “neratinib”, “tucatinib”, “pyrotinib”, “tyrosine kinase inhibitors”, “cardiotoxicity”, “cardiovascular toxicity”, “heart failure”, “left ventricular dysfunction”, “QT prolongation”, “arrhythmias”, “hypertension”, and “cardio-oncology”. Articles were considered eligible if they addressed at least one of the following topics: (i) molecular and pathophysiological mechanisms underlying cardiovascular toxicity of HER2-targeted agents or multikinase inhibitors; (ii) cardiovascular safety findings from phase II/III clinical trials; (iii) observational studies, registries, or real-world reports describing cardiovascular outcomes; and (iv) guideline recommendations or expert consensus documents relevant to surveillance and management in cardio-oncology. Priority was given to original research articles, meta-analyses, pivotal clinical trials, position statements, and international guidelines. Preclinical studies were included when they provided relevant mechanistic insight into myocardial injury, mitochondrial dysfunction, oxidative stress, endothelial damage, or electrophysiological alterations.

The selection of studies was based on their scientific relevance, methodological quality, and contribution to the understanding of cardiotoxicity profiles across different drug classes. Given the narrative nature of this review, no formal systematic review protocol, quantitative synthesis, or risk-of-bias assessment tool was applied. Instead, the available evidence was interpreted qualitatively and organized into thematic sections addressing cardio-oncology principles, mechanisms of toxicity, agent-specific cardiovascular profiles, real-world evidence, and practical management strategies (Table 1).

The final manuscript was designed to provide a comprehensive and clinically oriented synthesis of the literature, with particular emphasis on the heterogeneity of cardiovascular adverse events among monoclonal antibodies, antibody–drug conjugates, and tyrosine kinase inhibitors, as well as on the growing importance of multidisciplinary cardio-oncology care.

## 3. Cardio-Oncology: A Multidisciplinary Interface

Cardio-oncology has emerged as a distinct, rapidly evolving discipline at the intersection of cardiovascular medicine and oncology, aimed at preventing, diagnosing, and managing cancer therapy-related cardiovascular toxicity (CTR-CVT). This field is of increasing importance in the modern oncologic landscape, where the improved survival of patients receiving targeted and immunomodulatory therapies is counterbalanced by an increased burden of treatment-related cardiovascular complications. The paradigm shift toward chronic management of cancer, combined with the cardiometabolic vulnerability of many patients, has necessitated a proactive and collaborative approach to cardiovascular care in oncology settings. Cardio-oncology is defined as a multidisciplinary specialty involving cardiologists, oncologists, hematologists, radiologists, pharmacists, and nurse specialists, working together to optimize cardiovascular health in patients with cancer [10]. It encompasses the entire spectrum of cardiovascular complications, from asymptomatic subclinical dysfunction to overt heart failure, arrhythmias, thromboembolism, hypertension, and ischemic syndromes, related to conventional cytotoxic agents, targeted therapies (such as HER2 inhibitors and TKIs), radiotherapy, and newer agents including immune checkpoint inhibitors.

According to the 2022 ESC Guidelines on Cardio-oncology, developed in collaboration with the European Hematology Association (EHA), International Cardio-Oncology Society (ICOS), and other stakeholders, early identification of patients at risk is critical to reducing the morbidity and mortality associated with CTR-CVT [11]. The guidelines emphasize the importance of baseline cardiovascular risk assessment, which should include clinical evaluation, detailed history (including prior anthracycline exposure, radiation therapy, and cardiovascular disease), and baseline cardiac imaging. Stratification tools such as the HFA-ICOS proformas are recommended to categorize patients into low, medium, high, or very high cardiovascular risk prior to cancer therapy initiation. Cardiac imaging plays a central role in early detection and longitudinal monitoring of cardiotoxicity. Transthoracic echocardiography (TTE), with global longitudinal strain (GLS) and three-dimensional left ventricular ejection fraction (3D-LVEF), is the imaging modality of choice due to its wide availability and sensitivity to early myocardial changes. GLS allows detection of subclinical myocardial dysfunction before overt reduction in LVEF occurs [12] and is now considered a class I recommendation for surveillance in patients receiving potentially cardiotoxic therapy, including HER2-targeted agents.

Cardiac magnetic resonance imaging (CMR) offers superior reproducibility and tissue characterization, especially in cases of equivocal echocardiographic findings or suspected myocarditis and is recommended in selected cases [13]. Biomarkers, including cardiac troponins (high-sensitivity cTnI or cTnT) and natriuretic peptides (BNP or NT-proBNP), provide complementary information to imaging and have demonstrated prognostic value in identifying patients at risk of cardiotoxicity [14]. Troponin elevation during or after therapy is associated with subsequent development of left ventricular dysfunction, and serial measurements are advised particularly in high-risk populations.

**Figure 1:**
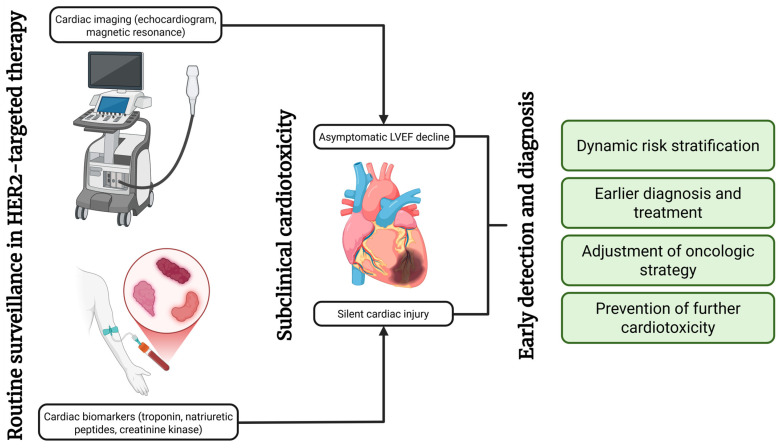
A surveillance strategy combining the use of cardiac imaging with biochemical testing using biomarkers associated with cardiac injury (troponins, creatinine kinase) and stress/heart failure (natriuretic peptides) allows for the detection of cardiotoxic effects of HER2-targeted therapies prior to the development of symptoms or clinical heart failure. Earlier detection of cardiotoxicity allows for a more nuanced and personalized management approach to the individual patient, including the possibility for risk stratification into low, moderate, severe categories, with implications for ongoing oncologic therapy and possible introduction of medications to treat and prevent further cancer therapy related cardiac dysfunction.

The combined use of imaging and biomarkers offers a synergistic approach to personalized monitoring strategies (Figure 1). Risk stratification models and surveillance algorithms are further guided by the type of anticancer agent, cumulative dose, patient-specific factors (age, comorbidities, prior cardiovascular disease), and treatment regimen. In patients receiving HER2-targeted therapies such as trastuzumab or TKIs like lapatinib and neratinib, a multimodal surveillance strategy with imaging every 3 months and biomarker assessments at each cycle is often warranted, especially in high-risk groups. Preventive strategies may include cardioprotective pharmacotherapy (e.g., beta-blockers, ACE inhibitors), lifestyle optimization, and in selected cases, temporary suspension or modification of cancer therapy [15].

The integration of cardio-oncology services into routine oncologic care has been shown to improve treatment adherence, reduce cardiac events, and facilitate timely oncologic decision-making [16,17,18,19,20,21,22]. Multidisciplinary cardio-oncology boards are increasingly recommended by ICOS and ESC as the standard of care for managing complex cases and facilitating shared decision-making, especially when balancing oncologic efficacy against cardiovascular risk. Cardio-oncology represents a critical interface in contemporary cancer care, necessitating structured collaboration, evidence-based surveillance protocols, and personalized therapeutic strategies. As cancer survivorship continues to rise, the implementation of cardio-oncology frameworks across institutions is imperative to minimize cardiovascular morbidity and ensure that the full benefit of novel anticancer therapies can be safely realized. These cardio-oncology principles are particularly relevant to HER2-targeted therapies, which have transformed outcomes in HER2-positive malignancies but remain among the best-characterized examples of targeted therapy-associated cardiac dysfunction. The need for baseline cardiovascular risk assessment, serial imaging, biomarker surveillance, and multidisciplinary decision-making is directly informed by the biological role of HER2 signaling in myocardial homeostasis. Accordingly, the following sections examine the mechanistic basis, clinical evidence, and management implications of cardiovascular toxicity associated with novel HER2-targeted agents.

## 4. Novel HER2-Targeted Agents

### 4.1. Mechanisms of Cardiotoxicity

Among contemporary targeted cancer therapies, HER2-directed agents provide a particularly instructive model of the balance between oncological efficacy and cardiovascular risk. HER2 (human epidermal growth factor receptor 2), a transmembrane tyrosine kinase receptor of the ErbB family, is most commonly recognized for its overexpression in certain subtypes of breast and gastric cancers, where it serves as a critical driver of tumor growth and proliferation [1,23]. However, HER2 also fulfills essential physiological roles in non-neoplastic tissues, particularly within the cardiovascular system. In adult cardiac tissue, HER2 expression is comparatively lower than in tumor cells, but it is functionally indispensable, especially under conditions of cellular stress or injury. In cardiomyocytes, HER2 does not function in isolation but instead forms heterodimers, primarily with HER4, in response to the activation of neuregulin-1 (NRG-1), a paracrine growth factor produced by cardiac endothelial cells [9]. This NRG-1/ErbB signaling axis is critical for maintaining myocardial structure and function. It promotes cardiomyocyte survival, modulates anti-apoptotic pathways (including PI3K/AKT and MAPK), sustains sarcomeric integrity, and facilitates the adaptive response to biomechanical stressors such as hypertension, ischemia, or volume overload [24]. Furthermore, this signaling cascade plays a central role in the regulation of calcium handling and mitochondrial function, both of which are vital for contractile performance and energetic homeostasis [25].

HER2 signaling is particularly important in scenarios involving myocardial injury, such as after myocardial infarction or in response to cytotoxic insults. In these settings, the NRG-1/HER2-HER4 interaction initiates reparative processes including cardiomyocyte proliferation, hypertrophic growth, and modulation of fibroblast activity to support tissue remodeling [7,8,26,27]. Disruption of this pathway through pharmacological blockade, as occurs with anti-HER2 therapies, effectively severs a key compensatory mechanism that the myocardium relies upon to survive and adapt. The inhibition of HER2 therefore deprives cardiomyocytes of an essential survival signal, increasing their vulnerability to oxidative stress, mechanical strain, and mitochondrial dysfunction. In addition to HER2/HER4 signaling, ErbB3/HER3 should also be considered within the broader HER-family signaling network. HER3 has impaired intrinsic kinase activity [28], but upon binding with NRG-1, forms highly active heterodimers with HER2, which are among the most potent signaling units within the ErbB receptor family [29]. This potency is partly explained by the presence of multiple binding sites for the p85 regulatory subunit of PI3K, enabling strong downstream activation of the PI3K/AKT pathway [30]. Although the NRG-1/HER2-HER4 axis remains the best characterized cardioprotective pathway, HER3 contributes to the wider regulation of cell survival, metabolic adaptation, and stress-response signaling [31]. From a cardio-oncology perspective, this reinforces the concept that HER2-targeted therapies may perturb not only isolated HER2 receptor activity, but an interconnected ErbB signaling network with relevance to cardiomyocyte resilience, mitochondrial homeostasis, and vulnerability to additional insults such as anthracyclines, hypertension, ischaemia, or ageing. Clinically, this disruption manifests as a decline in left ventricular systolic function, ranging from asymptomatic reductions in ejection fraction to overt heart failure. Recognition of this broader signaling context supports the need for baseline cardiovascular risk assessment, early detection of subclinical dysfunction and proactive cardioprotective measures in patients receiving HER2-directed therapies.

Notably, HER2-targeted therapy–associated cardiotoxicity is classically categorized as Type II cardiotoxicity, a phenotype that is distinct from the irreversible, dose-dependent injury seen with agents like anthracyclines (Type I) [32]. Type II cardiotoxicity is not associated with myocyte necrosis, but rather with functional impairment resulting from molecular interference in protective intracellular signaling. Importantly, this form of cardiotoxicity is often reversible upon cessation of the offending agent and appropriate cardiac management, particularly if identified and addressed early during therapy.

Data from animal and human cardiomyocyte models further suggest that the effects of HER2 inhibition may involve subcellular disruptions in mitochondrial bioenergetics and autophagic flux, impairing the cell’s ability to manage oxidative damage [25]. Additionally, HER2 appears to contribute to endothelial-cardiomyocyte crosstalk, and its blockade may also impair vascular responsiveness and myocardial perfusion at the microvascular level [33]. These findings support the hypothesis that HER2 signaling serves not merely as a developmental remnant in the adult heart, but as a dynamic and responsive system integral to cardiomyocyte homeostasis under both physiological and pathological conditions.

In summary, HER2 is not only a target in oncology but also a critical molecular safeguard in the myocardium. Interruption of this signaling, while therapeutically beneficial in HER2-overexpressing malignancies, can lead to significant, albeit often reversible, cardiac dysfunction. This mechanistic understanding underpins the need for vigilant cardiac monitoring and proactive management strategies in patients receiving HER2-targeted therapies, especially those with coexisting cardiovascular risk factors or prior exposure to cardiotoxic agents.

### 4.2. Direct Effects on Cardiomyocytes: Mitochondrial Dysfunction and Oxidative Stress

In addition to the interruption of essential survival signaling pathways, HER2-targeted therapies exert direct cytotoxic effects on cardiomyocytes, which contribute to their cardiotoxic potential, particularly under conditions of physiological or pharmacologic stress [34,35]. Among the most critical mechanisms implicated in this process is mitochondrial dysfunction, a hallmark of early cardiomyocyte injury observed in preclinical models. HER2 plays a regulatory role in mitochondrial integrity, biogenesis, and energy metabolism. When HER2 signaling is pharmacologically inhibited, particularly with monoclonal antibodies like trastuzumab, this balance is disrupted at several levels [36]. This leads to inefficient oxidative phosphorylation, diminished ATP synthesis, and leakage of electrons that facilitates excess production of reactive oxygen species (ROS) [34]. The resulting oxidative stress causes lipid peroxidation, protein denaturation, and mitochondrial DNA damage within cardiomyocytes, which are particularly vulnerable given their high metabolic demands and limited regenerative capacity [37]. Moreover, ROS-induced activation of pro-apoptotic factors, including Bax translocation and cytochrome c release, initiates the intrinsic (mitochondria-mediated) apoptotic cascade [25]. These intracellular events ultimately compromise cardiomyocyte viability and contribute to progressive contractile dysfunction, especially when superimposed on additional insults such as catecholamine surges, ischemia, or concurrent chemotherapy. The disruption of calcium homeostasis is another key pathological process driven by mitochondrial impairment. Dysfunctional mitochondria lose their ability to buffer intracellular calcium, leading to elevated cytosolic calcium levels, impaired excitation-contraction coupling, and increased susceptibility to calcium-triggered cell death [38]. This may manifest clinically as impaired systolic function, arrhythmias, or heightened vulnerability to adrenergic stimuli during stress or exertion.

Beyond trastuzumab, newer HER2-targeted agents such as antibody–drug conjugates (ADCs) introduce additional layers of complexity. ADCs like trastuzumab emtansine (T-DM1) and trastuzumab deruxtecan (T-DXd) are engineered to selectively deliver cytotoxic payloads to HER2-expressing tumor cells [39]. However, despite the targeted design, there is potential for off-target cardiac toxicity [40]. T-DM1 links trastuzumab to the microtubule inhibitor DM1, which, although intended to remain intracellularly confined within HER2-overexpressing cancer cells, may inadvertently be internalized by HER2-expressing cardiomyocytes or exert paracrine effects if prematurely released into the systemic circulation [41]. Similarly, T-DXd includes a topoisomerase I inhibitor payload with a higher drug-to-antibody ratio and a cleavable linker [42], which raises concerns about systemic diffusion of the cytotoxic agent, potentially leading to DNA damage and apoptosis in cardiac tissue even at low HER2 expression levels. Moreover, T-DM1 has been shown in a preclinical study to impair mitochondrial function and augment ROS production [43]. These findings raise the possibility that cumulative mitochondrial injury may occur with sequential or prolonged HER2-directed ADC therapy, particularly in patients with underlying mitochondrial dysfunction due to age, metabolic disease, or prior anthracycline exposure. Importantly, the direct cardiotoxic effects of HER2-targeted therapies are not uniformly expressed across all patients, underscoring the role of individual susceptibility. Factors such as baseline mitochondrial reserve, oxidative stress burden, and genetic polymorphisms in antioxidant defense mechanisms likely modulate the degree of mitochondrial injury and clinical expression of cardiotoxicity. For example, patients with diabetes or obesity may exhibit pre-existing mitochondrial compromise, rendering them more vulnerable to drug-induced injury.

The risk of HER2 antagonist-induced cardiotoxicity is substantially increased by prior or concomitant anthracycline exposure [44]. Anthracyclines cause cumulative myocardial injury through several interrelated mechanisms, including reactive oxygen species generation, mitochondrial dysfunction, lipid peroxidation, DNA damage, inhibition of topoisomerase IIβ, and activation of cardiomyocyte apoptosis [45]. This pattern of injury is classically considered dose-dependent and may lead to irreversible myocyte loss. In contrast, HER2-targeted cardiotoxicity is often mediated by interruption of adaptive cardioprotective signalling rather than direct myocyte necrosis. The interaction between these two mechanisms can be understood as a “dual-hit” model: anthracyclines first induce structural and mitochondrial injury, while subsequent HER2 blockade impairs neuregulin-1/ErbB-mediated survival, repair, and stress-response pathways that would otherwise help maintain myocardial function. As a result, cardiomyocytes previously exposed to anthracyclines become more vulnerable to HER2 inhibition, with a greater likelihood of left ventricular ejection fraction decline and symptomatic heart failure. Clinically, this interaction was evident in early adjuvant trastuzumab trials [44], in which cardiac events were more frequent when trastuzumab was administered sequentially after anthracycline-based chemotherapy, supporting the need for careful baseline risk assessment and serial cardiac surveillance in patients receiving both treatment classes.

**Figure 2:**
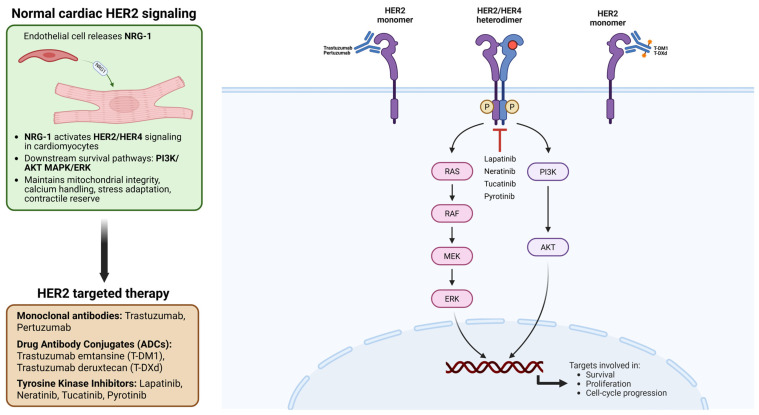
Mechanistic pathways involved in HER2 signalling. In the healthy myocardium, neuregulin-1 (NRG-1) released by cardiac endothelial cells activates HER2/HER4-mediated cardioprotective signalling in cardiomyocytes. HER2-targeted monoclonal antibodies and antibody–drug conjugates disrupt these pathways by preventing dimerization and downstream intracellular signalling pathways. Antibody–drug conjugates may also contribute additional payload-related injury. Tyrosine kinase inhibitors disrupt cell signalling pathways by binding to intracellular ATP-binding sites of HER receptors.

In conclusion, HER2-targeted therapies, especially trastuzumab and next-generation ADCs, exert direct toxic effects on cardiomyocytes through mechanisms that converge on mitochondrial dysfunction, oxidative stress, and calcium mishandling. These processes can lead to energetic failure, activation of apoptosis, and impaired contractile performance (Figure 2). Understanding these molecular underpinnings is critical for developing targeted cardioprotective interventions, identifying high-risk patients, and guiding the design of next-generation HER2-targeted agents with reduced cardiac liability.

### 4.3. Clinical Trial Data: Trastuzumab, Pertuzumab, T-DM1, Trastuzumab Deruxtecan

The reported incidence of trastuzumab-associated cardiotoxicity varies across studies, reflecting heterogeneity in treatment setting, prior anthracycline exposure, baseline cardiovascular risk, definitions of cardiotoxicity, and intensity of cardiac surveillance. In early metastatic breast cancer studies combining trastuzumab with chemotherapy (particularly those involving anthracycline-containing regimens), the risk of cardiac dysfunction was relatively high, establishing the clinical importance of HER2-related cardiotoxicity [46,47,48,49,50,51,52]. In the pivotal NSABP B-31 and NCCTG N9831 trials, the addition of trastuzumab to chemotherapy increased cardiac events from ~1% to 4–5% [47]. Non-anthracycline and de-escalated regimens generally showed lower rates of clinically significant cardiotoxicity [48,49,53,54,55,56,57,58]. Long-term follow-up suggests that most events occur during treatment or shortly thereafter and are often at least partially reversible, although persistent dysfunction can occur [59,60,61,62,63]. Therefore, trastuzumab cardiotoxicity should be considered a risk-modified phenomenon rather than a single fixed incidence estimate.

Overall, rates of asymptomatic left ventricular ejection fraction (LVEF) decline are estimated to be 5–10% of patients, with symptomatic heart failure in 1–4%. The risk increases significantly with prior anthracycline exposure and older age.

Pertuzumab, when combined with trastuzumab and a taxane did not result in a substantial increase in cardiotoxicity rates [64,65,66,67,68,69]. In CLEOPATRA [64], LV dysfunction was reported in 4%, with 1% having symptomatic LV dysfunction. In the APHINITY trial, symptomatic heart failure was 0.6%, all occurring in patients treated with anthracyclines; asymptomatic or mildly symptomatic LVEF decline was seen in 3% of patients [69].

T-DM1 (trastuzumab emtansine), by linking trastuzumab to a cytotoxic agent (DM1), maintains anti-HER2 activity while demonstrating a lower incidence of cardiac dysfunction compared to trastuzumab monotherapy in many studies [70,71,72,73,74], including the EMILIA [70] and KATHERINE [74] trials, with prevalence of asymptomatic LVEF decline ranging from 0.4–3%. However, cardiotoxicity remains a concern, particularly in patients with pre-existing cardiac disease or prolonged HER2 blockade.

Trastuzumab deruxtecan (T-DXd), a next-generation ADC, has raised questions regarding long-term cardiotoxicity due to its higher drug-to-antibody ratio and potential for systemic payload exposure. In patients receiving T-DXd at a dose of 5.4 mg/kg, LVEF decline was seen in 4.6% of patients, of which 0.6% were severe; in patients administered 6.4 mg/kg, LVEF decline was reported in 2%, with no events of heart failure [75,76,77,78,79,80,81,82,83,84]. Ongoing surveillance is warranted due to the novelty of the agent and prolonged treatment durations in metastatic settings.

Real-world evidence regarding the incidence of cardiotoxicity with ADC treatments is not yet as robust as for other HER2 directed treatments. Despite this, emerging evidence suggests lower rates of cardiotoxicity with T-DM1, with comparable incidence for T-DXd and standard trastuzumab regimens [85].

### 4.4. Real-World Registries and Observational Studies

Data from real-world cohorts complement clinical trial findings and highlight patient populations at increased risk for cardiotoxicity, including older adults, individuals with baseline cardiac dysfunction, and those receiving cumulative anthracycline exposure. Registries such as the SAFE-HEaRt study have explored the feasibility of continuing HER2-targeted therapies in patients with reduced LVEF under cardiology co-management [86]. Additionally, observational studies have noted underutilization of cardioprotective strategies (e.g., beta-blockers, ACE inhibitors) in clinical practice, suggesting gaps between guidelines and real-world care [87,88]. Longitudinal data also indicate that even mild LVEF declines may carry prognostic significance, particularly in the metastatic setting [89,90].

## 5. Tyrosine Kinase Inhibitors (TKIs)

### 5.1. Mechanisms of Cardiotoxicity

#### 5.1.1. Multi-Kinase Inhibition and Cardiovascular Off-Target Effects

HER2-targeted TKIs, such as lapatinib, neratinib, tucatinib, and pyrotinib, vary widely in selectivity. Lapatinib [91] and neratinib [92] are dual inhibitors of EGFR and HER2, whereas tucatinib [93] is more HER2-specific, and pyrotinib [94] irreversibly inhibits pan-ErbB family members. Beyond HER2, many TKIs also target off-target kinases located in cardiovascular tissues, such as VEGFR, Src, or PDGFR, which can influence vascular tone, endothelial integrity, and myocardial cell signaling [95]. These off-target effects can lead to endothelial dysfunction, vasoconstriction, or disruption of cardiomyocyte survival signals, increasing cardiovascular risk beyond pure HER2 blockade [96].

#### 5.1.2. Hypertension, Endothelial Dysfunction, Electrophysiological Changes

Hypertension is among the most common cardiovascular toxicities observed with TKIs, linked to impaired nitric oxide synthesis, increased vascular resistance, and microvascular rarefaction [97,98]. Endothelial dysfunction resulting from VEGFR inhibition has been documented in TKI-treated oncology patients, and although HER2-specific TKIs do not target VEGFR directly, their multi-kinase activity can still perturb endothelial signaling [95,96,99,100,101]. Additionally, several TKIs, most notably lapatinib, carry a risk, albeit low, of QTc prolongation and arrhythmias due to interference with cardiac ion channels [102,103,104]. The combination of hypertension, vascular changes, and minor electrophysiological effects creates a cardiovascular milieu that may predispose to heart failure or ischemic events, especially in vulnerable patients.

#### 5.1.3. Clinical Evidence and Real-World Data

##### Lapatinib

A meta-analysis of 45 studies in breast cancer patients reported an overall incidence of cardiac adverse events (AEs) of 2.7% with lapatinib, including 1.6% left ventricular dysfunction and 2.2% LVEF decrease [105]. A pooled analysis of 3689 patients from 44 trials found low rates of cardiotoxicity for lapatinib, with asymptomatic LVEF decline reported in 1.3% of patients and symptomatic HF in 0.3% [106]. Though lower than rates seen with trastuzumab, this is notable given lapatinib’s off-target kinase profile. A meta-analysis comparing lapatinib and trastuzumab found a lower incidence of cardiac AEs with lapatinib, including heart failure and LVEF decline [107]. QT prolongation has also been observed in an early pharmacokinetic trial, cautioning against use in patients with electrolyte imbalance or congenital long-QT syndrome [108]. In a clinical trial, patients treated with lapatinib/capecitabine had a reported prevalence of QT prolongation of 3.9% with an incidence of cardiac arrythmias of 3.5% [109]. In real-world practice, those with pre-existing cardiac risk factors or on other QT-prolonging medications warrant closer ECG and echocardiographic follow-up [11].

##### Neratinib

Neratinib is a covalent dual EGFR/HER2 inhibitor with a low incidence of cardiotoxicity, with no significant cardiotoxicity reported in early trials [109,110,111,112]. Diarrhea and hepatotoxicity dominate its toxicity profile. Cardiovascular AEs have been minimal, though formal QT analyses from early-phase trials are limited and long-term cardiac monitoring data remain sparse. A trial of neratinib/capecitabine reported an incidence of cardiac arrythmias of 3.3 and of QT prolongation of 2.3% [109].

##### Tucatinib

As a highly selective, reversible HER2 inhibitor with little off-target kinase activity, tucatinib has demonstrated no significant cardiotoxicity signal in both clinical trials and post-marketing surveillance [113]. The HER2CLIMB trial reported excellent safety, with rare LVEF decline or ECG abnormalities [114]. However, hypertension remains a theoretical risk, so periodic blood pressure assessments are advised.

##### Pyrotinib

Pyrotinib is a novel irreversible pan-ErbB TKI showing promising efficacy, especially in brain metastatic disease. Cardiovascular AEs have been rare in both phase I/II studies and larger trials [115,116,117,118,119]. An analysis of cardiotoxicity from the PLEHERM study found that lipid abnormalities were the main form of cardiotoxicity, with no cases of LVEF decline or heart failure [120]. An efficacy and safety study of 113 patients reported no significant increase in LVEF decline or hypertension compared to controls [121]. Nevertheless, the irreversible pharmacology merits longitudinal monitoring, particularly in patients with vascular comorbidities. A study aimed at assessing the cardiac effects of pyrotinib, EARLY-MYO-BC was established in 2023 and is ongoing [122].

#### 5.1.4. Incidence Rates, Risk Factors, and Outcomes

Meta-analytic and clinical trial data suggest differing cardiotoxic profiles across TKIs, with lapatinib having the highest risk of cardiotoxicity and relatively few reports of cardiotoxicity with the other agents (Table 1).

**Table 1:** Inclusion and exclusion criteria for study selection.

Category	Inclusion Criteria	Exclusion Criteria
**Study type**	Original research articles, randomized controlled trials, phase II/III clinical trials, meta-analyses, systematic reviews, observational studies, registries, international guidelines, and expert consensus statements	Case reports (unless highly relevant), editorials, letters without original data, conference abstracts lacking full data
**Population**	Adult patients receiving HER2-targeted therapies or tyrosine kinase inhibitors for oncologic indications	Pediatric populations; non-oncologic use of HER2-targeted therapies or TKIs
**Interventions**	HER2-targeted monoclonal antibodies, antibody–drug conjugates (ADCs), and tyrosine kinase inhibitors (e.g., trastuzumab, pertuzumab, T-DM1, T-DXd, lapatinib, neratinib, tucatinib, pyrotinib)	Studies not involving HER2-targeted agents or relevant TKIs
**Outcomes**	Cardiovascular toxicity outcomes, including left ventricular dysfunction, heart failure, arrhythmias, QT prolongation, hypertension, endothelial dysfunction, and related biomarkers	Studies not reporting cardiovascular outcomes
**Mechanistic insight**	Preclinical or translational studies elucidating molecular mechanisms of cardiotoxicity (e.g., mitochondrial dysfunction, oxidative stress, signaling pathways)	Studies lacking mechanistic or clinical relevance to cardiotoxicity
**Language**	Articles published in English	Non-English publications
**Timeframe**	No strict time restriction; emphasis on contemporary and clinically relevant literature	Obsolete or outdated studies with limited relevance to current clinical practice

Lapatinib is the most studied of the aforementioned TKIs, with the largest and most robust data on cardiotoxicity. The other TKIs mentioned are more novel, with fewer data available. Initial studies indicate very low incidence of cardiotoxicity; however, long-term data and larger studies and meta-analyses are awaited.

Risk stratification hinges on patient-level factors, age, hypertension, baseline LVEF, previous anthracycline exposure, and the pharmacodynamics of each TKI. ECG baseline and periodic checks, as well as blood pressure monitoring, should be tailored to each agent’s risk profile.

Long-term follow-up data on HER2-targeted TKIs remain scarce, particularly beyond five years after therapy initiation. Available evidence, especially from cohorts treated with lapatinib in combination with chemotherapy or trastuzumab, suggests that the majority of cardiotoxic events tend to occur early, typically within the first few months of treatment [101,109,123,124,125]. Importantly, more than 80% of these cardiac events, primarily asymptomatic declines in left ventricular ejection fraction (LVEF), are reversible upon prompt discontinuation of the TKI. Despite this apparent reversibility, lingering effects such as persistent hypertension, hyperlipidemia or subclinical diastolic dysfunction, especially in older survivors, may remain under-recognized and require further prospective investigation. These late-onset or smoldering cardiovascular changes may subtly impair long-term cardiac reserve and potentially contribute to cumulative cardiovascular morbidity.

### 5.2. Prevention, Reversibility and Management Strategies

Prevention and management of HER2-targeted therapy- and TKI-associated cardiotoxicity require an integrated cardio-oncology approach that begins before cancer treatment is initiated. Baseline cardiovascular risk assessment should include a detailed history of cardiovascular disease and risk factors, prior anthracycline or mediastinal radiotherapy exposure, blood pressure assessment, ECG, cardiac biomarkers where appropriate, and baseline transthoracic echocardiography including LVEF and, where available, global longitudinal strain. Patients at high or very high baseline cardiovascular risk should be considered for early cardio-oncology referral before treatment initiation.

Primary prevention focuses on optimisation of modifiable cardiovascular risk factors, including hypertension, diabetes, dyslipidaemia, obesity, smoking, renal dysfunction, and pre-existing heart failure. In selected high-risk patients, cardioprotective pharmacotherapy with ACE inhibitors, angiotensin receptor blockers, beta-blockers, or mineralocorticoid receptor antagonists may be considered, particularly when there is previous anthracycline exposure, borderline baseline LVEF, abnormal GLS, or elevated cardiac biomarkers. Unlike dexrazoxane for the prevention of anthracycline related cardiotoxicity, no specific treatments or preventative medications exist for cardiotoxicity related to HER2 treatments.

One of the hallmarks of HER2-targeted cardiotoxicity is reversibility [126,127]. In most cases, LVEF improves partially or fully upon interruption of HER2 therapy, especially if detected early and treated promptly. Cardiac dysfunction is more likely to recover if ejection fraction does not fall below 40%, and recovery is typically observed within 3–6 months.

Management includes regular cardiac monitoring with echocardiography or MUGA scans, especially in the adjuvant setting. Guidelines recommend cardiac imaging every 3 months during trastuzumab-based therapy [11]. In patients with asymptomatic LVEF decline, initiation of cardioprotective agents such as beta-blockers or ACE inhibitors (e.g., carvedilol, enalapril) can facilitate recovery and potentially allow for continuation of HER2-targeted therapy [15,89]. Importantly, the latest ESC guidelines suggest that patients with symptomatic and moderate-severe asymptomatic CTRCD should be started on heart failure therapy [11].

For TKI-associated toxicity, management should be tailored to the dominant cardiovascular phenotype. Blood pressure should be measured regularly, and hypertension should be treated promptly. For agents associated with QT prolongation, baseline and interval ECG monitoring should be considered, particularly in patients with electrolyte abnormalities, renal impairment, concomitant QT-prolonging drugs, or prior arrhythmia. Correction of hypokalaemia, hypomagnesaemia, and other reversible risk factors is essential. New arrhythmias, persistent QTc prolongation, uncontrolled hypertension, or suspected ischaemia should prompt specialist cardio-oncology or cardiology review.

A rigorous baseline cardiac evaluation is essential before initiating any HER2-targeted TKI. This assessment should include both an electrocardiogram (ECG) and a transthoracic echocardiogram to document pre-treatment LVEF and screen for occult conduction abnormalities or structural heart disease. While current ESC guidelines suggest 3-monthly echocardiography in all patients treated with HER2-targeted therapies, the frequency and intensity of subsequent monitoring should be guided by the specific agent used and the patient’s baseline cardiovascular risk profile. For patients receiving lapatinib, echocardiographic evaluation is recommended at least every three to six months during treatment, with periodic ECG monitoring and electrolyte assessment in those with known or suspected risk factors for QTc prolongation. In contrast, for more HER2-selective agents such as neratinib, tucatinib, or pyrotinib, the surveillance strategy may be less intensive. In these cases, an echocardiogram at baseline, followed by a repeat scan at six months and annually thereafter, may be sufficient, provided the patient remains clinically stable. Blood pressure should be measured at every clinical visit, regardless of the agent used, given the well-documented hypertensive potential of many TKIs [128].

If hypertension develops, it should be managed promptly and aggressively according to standard cardio-oncology guidelines, with preference for agents that also confer cardioprotective effects, such as ACE inhibitors or beta-blockers. If a patient develops a significant drop in LVEF, defined as a decline of ≥10% from baseline to a value <50% [129], a referral to cardio-oncology is warranted for further evaluation and management. Additional red flags for specialist referral include persistent hypertension despite dual antihypertensive therapy, the development of new arrhythmias, or evidence of QT interval prolongation on ECG.

Should clinically significant cardiotoxicity emerge, temporary interruption or dose reduction of the TKI may be necessary. In many cases, once cardiac function has stabilized, often with the aid of cardioprotective medications, a cautious rechallenge can be considered, especially in patients who derive substantial oncologic benefit from continued HER2 inhibition. The decision to resume therapy must be individualized, balancing oncologic efficacy with cardiovascular safety, and ideally guided by close collaboration between oncology and cardio-oncology teams.

In summary, HER2-targeted TKIs present a generally favorable cardiac safety profile compared to monoclonal antibodies, though risks remain agent-specific. Mechanisms and phenotypes of toxicity are summarized in Table 2 and Table 3. Lapatinib poses modest LVEF and QTc risks; neratinib and pyrotinib exhibit low cardiotoxicity, and tucatinib appears especially safe. Vigilant cardiac monitoring, control of risk factors including hypertension and lipid control, and proactive co-management remain essential. Long-term registry data are needed to define late-onset effects and refine personalized follow-up protocols. Multidisciplinary collaboration between oncology and cardio-oncology is essential for individualized risk stratification and monitoring, particularly in patients receiving novel ADCs or prolonged HER2 blockade.

## Figures and Tables

**Table 2:** Summary of HER2 targeted therapies and their cardiotoxic effects.

Agent	Drug Class	Main Cardiovascular Toxicities	Reversibility
Trastuzumab	Anti-HER2 monoclonal antibody	LVEF decline (5–10%); symptomatic HF (1–4%)	Often reversible if detected early
Pertuzumab	Anti-HER2 monoclonal antibody targeting dimerization domain	LVEF decline (1–4%); symptomatic HF (~1%)	Usually reversible
T-DM1	HER2 antibody–drug conjugate; trastuzumab linked to DM1	LVEF decline (1–3%); rare symptomatic HF events	Usually reversible or partially reversible
T-DXd	HER2 antibody–drug conjugate; trastuzumab linked to topoisomerase I inhibitor	LVEF decline (2–4%); severe LVEF decline (<1%)	Appears often reversible, but evidence less mature than for trastuzumab
Lapatinib	Dual EGFR/HER2 inhibitor	LVEF decline(1–2%), symptomatic HF (<0.5%), QT prolongation (rare), arrhythmia (rare)	Most events appear reversible with interruption
Neratinib	Irreversible dual EGFR/HER2 inhibitor	Minimal toxicity; arrythmia (~3%), QT prolongation (~2%)	Appears largely reversible, though long-term cardiac data are sparse
Tucatinib	Highly selective HER2 inhibitor	Minimal toxicity; rare cases of LVEF decline; rare cases of ECG abnormalities	Available data suggest favorable reversibility
Pyrotinib	Irreversible pan-ErbB inhibitor	Lipid abnormalities; limited medium-long term data	Appears favorable, though evidence remains immature

**Table 3:** Summary of HER2 therapies and their dominant mechanism/phenotype.

Drug Group	Dominant Mechanism	Dominant Phenotype
Monoclonal antibodies	HER2 cardioprotective pathway interruption	LVEF decline/Heart failure
Antibody Drug Conjugates (ADCs)	HER2 blockade + payload-related cellular injury	LVEF decline, possible cumulative toxicity
Tyrosine Kinase Inhibitors (TKIs)	Multikinase off-target vascular/electrical effects	Hypertension, QT prolongation, arrhythmia, hyperlipidemia

## Data Availability

No new data were created or analyzed in this study. Data sharing is not applicable to this article.
